# Necrotizing Sialometaplasia: A Diagnostic Challenge to Oral Physicians

**DOI:** 10.7759/cureus.33122

**Published:** 2022-12-30

**Authors:** Nivedha Senthilnathan, Karthik Rajaram Mohan, Saramma Mathew Fenn, Ravikumar Pethagounder Thangavelu

**Affiliations:** 1 Oral Medicine and Radiology, Vinayaka Mission's Sankarachariyar Dental College, Vinayaka Mission's Research Foundation, Salem, IND

**Keywords:** oral malignancy, salivary gland disorders, hard palate, cone-beam computed tomography (cbct), necrotizing sialometaplasia

## Abstract

Necrotizing sialometaplasia is a rare, reactive, self-limiting disorder affecting a minor salivary gland that clinically mimics malignancy. Chronic smoking, alcohol use, trauma to the hard palate caused by local anesthetic injection due to the vasoconstrictive action of adrenaline in local anesthetic, topical application of nonsteroidal anti-inflammatory drugs like flurbiprofen spray used in bronchial asthma, oral intubation procedures for general anesthesia, ill-fitting dentures, bulimia nervosa, and minor salivary gland tumors are some of the contributing factors linked to its development. In this article, we discuss a rare, reactive, self-limiting condition affecting minor salivary gland, necrotizing sialometaplasia that occurred on the right posterolateral hard palate region in a 57-year-old male chronic smoker, diagnosed by oral medicine specialist by clinical findings and radiological evaluation by cone beam computed tomography that healed rapidly in three days by itself without any treatment, that prevented unwanted biopsy or surgery.

## Introduction

Necrotizing sialometaplasia is a rare, benign, self-limiting, reactive inflammatory disorder of the minor salivary gland mimicking a malignancy. The etiology is unknown and controversial. Some authors believed a local ischemic event caused it. Usually, it appears on the hard palate. However, the lesion was also reported on the floor of the mouth, tongue, lips, retromolar trigone, and hypopharynx area. Initially, it starts as a localized swelling followed by the appearance of a tender erythematous nodule that later breaks down into an ulcer with a non-indurated necrotic base. The patient affected by necrotizing sialometaplasia is usually painless and asymptomatic or gives a history of discomfort due to moderate dull pain in the affected region on the hard palate. The lesion often tends to occur following localized ischemia caused by infiltration of local anesthesia on the hard palate on the injection site area weeks after a dental procedure.

## Case presentation

**Figure 1:**
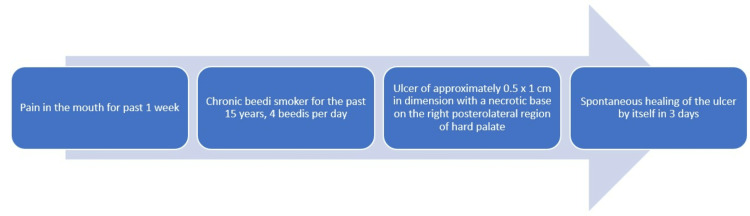
Brief timeline of history

A 57-year-old male reported to our department with a chief complaint of mouth pain for the past week. Extraoral examination revealed no cervical lymphadenopathy. The patient did not have diabetes. The patient is a chronic bidi smoker who smoked about four bidis per day for 15 years. A brief timeline of history is described in Figure 1.

**Figure 2:**
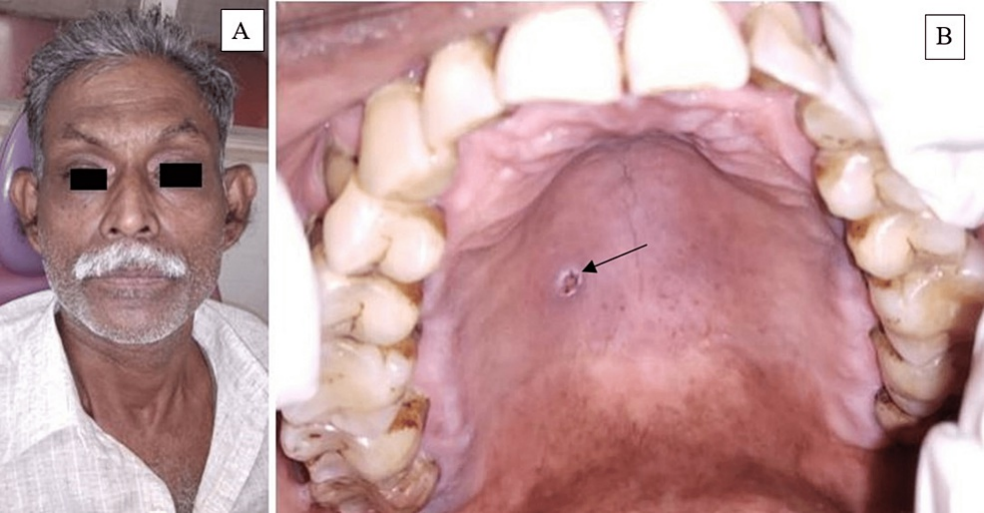
A. Extraoral examination did not reveal any facial asymmetry. B. Intraoral examination revealed an ulcer of dimension about 0.5 x 1 cm in diameter with a necrotic base on the right posterolateral region on the hard palate.

The extraoral examination did not reveal any facial asymmetry (Figure 2A). Intraoral soft tissue examination revealed an ulcer of approximately 0.5 x 1cm in size, oval in shape, brown discolored necrotic base on the right posterolateral region of the hard palate (Figure 2B).

**Figure 3:**
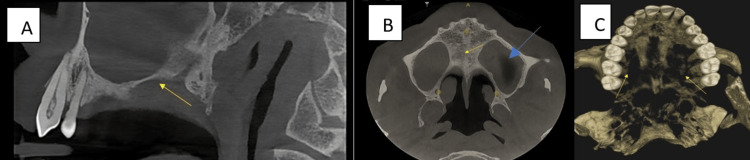
CBCT A. Sagittal section revealed no erosive changes and intact floor of the maxillary sinus (yellow arrow). B. Axial section revealed increased mucosal thickening within the left maxillary sinus (blue arrow) and no erosive changes on the hard palate (yellow arrow). C. 3D-reconstructed CBCT revealed a bone loss in the posterior maxillary quadrants

On palpation, the ulcer was non-tender and base non-indurated. Correlating the history of chronic bidi smoking four bidis per day for the past 15 years and intraoral clinical findings, a provisional diagnosis of necrotizing sialometaplasia was made. The differential diagnoses were soft palate subacute necrotizing sialadenitis, mucormycosis, traumatic ulcerative granuloma with stromal eosinophilia (TUGSE), cancrum oris (noma), minor salivary gland tumors like squamous cell carcinoma, mucoepidermoid carcinoma, adenoid cystic carcinoma, Warthin’s tumor. The sagittal section (CBCT) did not show any erosive changes on the floor of the maxillary sinus. (Figure 3A). The axial section revealed mucosal thickening within the left maxillary sinus and no erosive changes in the hard palate (Figure 3B). 3D-reconstructed CBCT image revealed a bone loss in the right and left posterior maxillary teeth due to chronic periodontal disease (Figure 3C).

**Figure 4:**
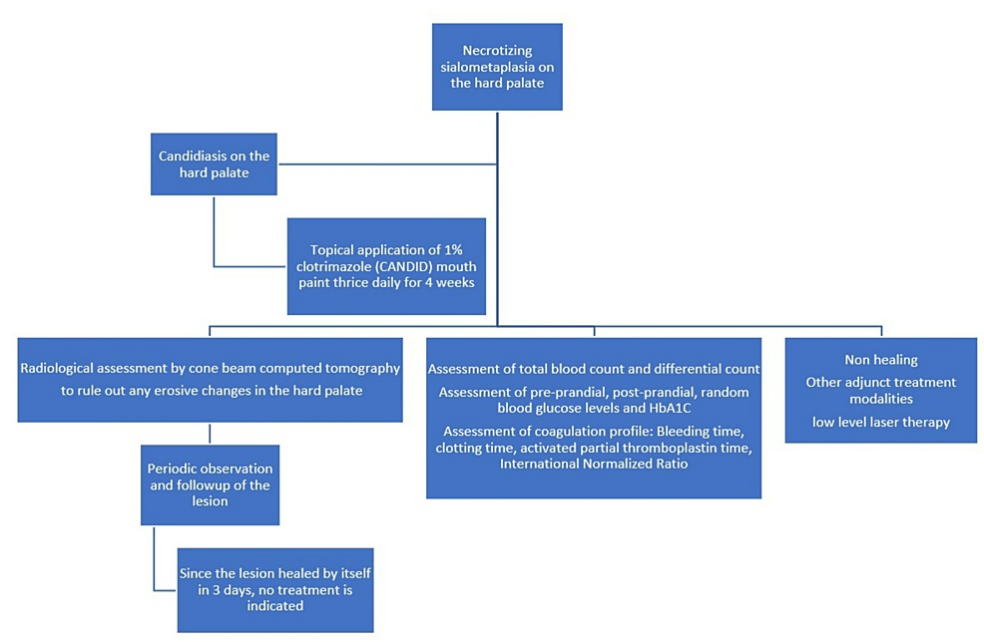
Diagnostic workup of necrotizing sialometaplasia on the hard palate

Therapeutic intervention: The therapeutic intervention for necrotizing sialometaplasia and candidiasis on the hard palate is described in Figure 4.

**Figure 5:**
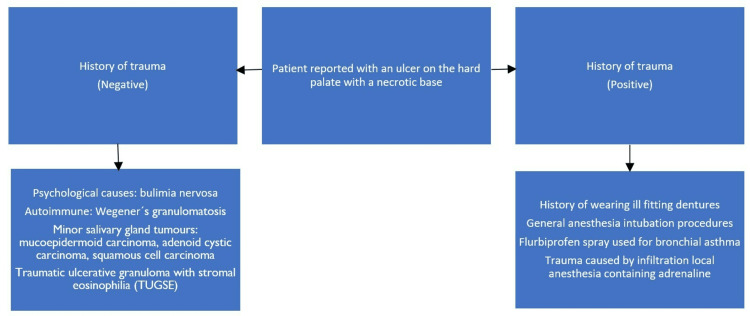
Diagnostic assessment of Necrotizing sialometaplasia on the hard palate

Diagnostic assessment: The laboratory investigation of total blood count and differential count, pre-prandial, post-prandial, random blood glucose level, and HbA1C were within biological ranges. Bleeding time, clotting time, and prothrombin time was also assayed and showed the absence of any coagulopathy. The diagnostic assessment for necrotizing sialometaplasia is described (Figure 5).

Correlating the chief complaint, history, and clinical and radiographic findings, a final diagnosis of necrotizing sialometaplasia was made. The recent protocol for the management of necrotizing sialometaplasia is described (Table 1).

**Table 1:** Recent protocol for management of necrotizing sialometaplasia

Size of the Lesion	Treatment Modalities	Technique	Mechanism of Action	Healing period
0.5 – 1 cm in diameter	No treatment	Wait and watch. Close observation and follow-up.	Healing is self-limited. Heals by itself.	No treatment can disappear in three days- one week.
1.5 cm in diameter	Photobiomodulation therapy or low-level laser therapy or bio-modulation	Red light of wavelength 660 nm, 30 Mw of power, a fluence of 1.1 J/cm^2^, irradiation time of 1 minute 40 seconds.	Accelerates wound healing by promoting inflammatory responses by stimulating oxidative Phosphorylation by ATP in Mitochondria (Powerhouse of the cell). It also aids in controlling secondary infection by decreasing the pathogens in the area.	a) Non-invasive b) shorter healing time 4-10 weeks.
3 x 4 cm	Surgical debridement by curettage followed by saline irrigation rotated pedicled palatal flap	Subperiosteal dissection and rotated pedicled palatal flap.	The area healed by epithelization.	Longer healing period. Approximately three months.

**Figure 6:**
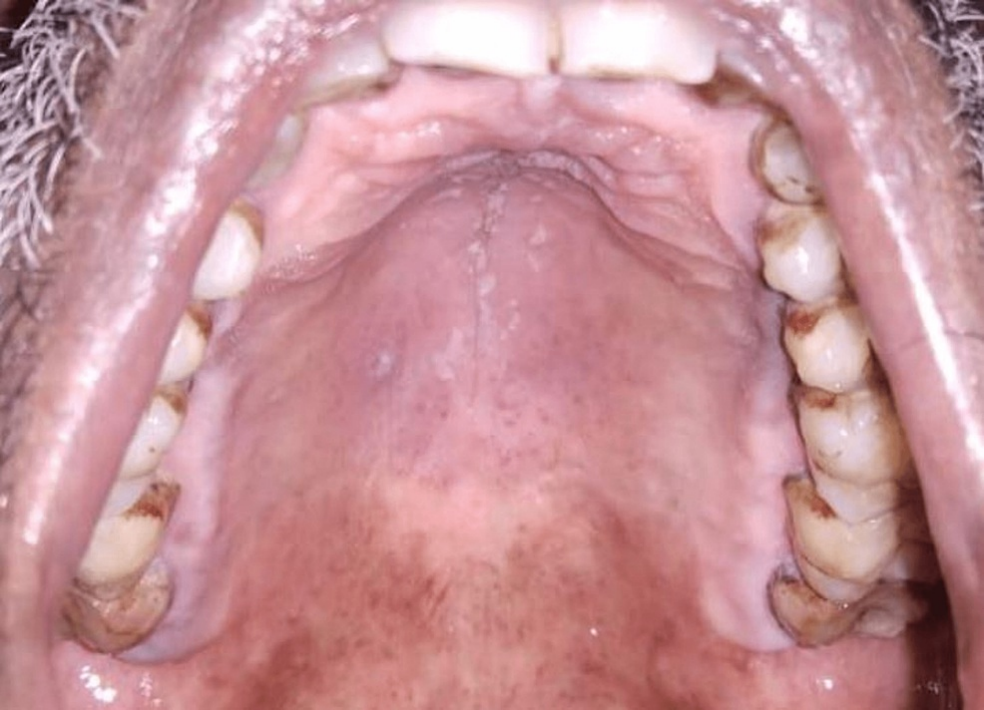
Follow-up after three days showed resolution of the lesion on the hard palate

Follow-up and outcome: The patient followed up after three days and revealed complete resolution of the lesion on the hard palate. Only discrete curdy white precipitates were scattered on the hard palate region, suggestive of candidiasis, for which he was prescribed topical 1% clotrimazole mouth paint application thrice daily for four weeks (Figure 6).

## Discussion

Necrotizing sialometaplasia is a rare, benign, self-limiting, reactive inflammatory disorder of the salivary gland tissue that clinically mimics a malignancy. Necrotizing sialometapalsia was first clinically reported by Abrams in 1973. The etiology is unknown and controversial. Some attribute its formation due to chronic smoking [1]. Some authors attribute the cause to local trauma from the injection site during infiltration anesthesia in the vicinity of the hard palate before performing a dental extraction procedure. The adrenaline in local anesthetic causes localized vasoconstriction and ischemia, which results in its formation. [2] Trauma from intubation procedure for general anesthesia also can result in necrotizing sialometaplasia, bulimia nervosa, an eating disorder with marked binge eating followed by purging [3,4]. Gatti A et al. described a necrotizing sialometaplasia caused by excessive use of the nonsteroidal anti-inflammatory drug Flurbiprofen oral spray that contained alcohol six times once a day for five weeks for prevention of sore throat [5]. Senapati S et al. reported its occurrence due to immune-mediated vasculitis such as granulomatosis with polyangiitis Wegener’s disease [6]. Trauma from the use of dentures, consumption of alcohol, and habit of tobacco use were also considered predisposing factors for necrotizing sialometaplasia. The most predominant site of occurrence was the hard palate region, where there is abundant minor salivary gland tissue. Lesions were also reported on the retromolar trigone, lip, hypopharynx, and tonsillar pillar. Usually, the lesions appear unilaterally. Kandula S et al. (2016) reported a bilateral occurrence of the lesion on the hard palate [7]. The lesion initially starts as an erythematous nodule or swelling, which breaks and transforms into an ulcer with a necrotic base. The patient gives a history of rapid onset, usually a day or two, but complaints of pain in the region. The clinical differential diagnosis includes soft palate subacute necrotizing sialadenitis (SANS), mucormycosis, squamous cell carcinoma, mucoepidermoid carcinoma, adenoid cystic carcinoma, and Warthin’s tumour. Soft palate subacute necrotizing sialadenitis involves the soft palate, uvula, ventral surface of the tongue, buccal mucosa, and lip. Necrotizing sialometaplasia can affect major salivary glands, the nasal cavity, the maxillary sinus, and the larynx. Necrotizing sialometaplasia can induce the squamous metaplastic transformation in acinar cells, but subacute necrotizing sialadenitis of the soft palate does not [8]. Mucormycosis can clinically present as a necrotic ulcer on the hard palate but usually occurs in patients with uncontrolled diabetes mellitus or long-term antibiotic, steroid, or antifungal therapy. The patient who reported to our department was not a diabetic and was not under any of the above medications, which can compromise the immune system in the long term. Traumatic ulcerative granuloma with stromal eosinophilia (TUGSE) usually occurs on the tongue following the onset of trauma. The patient, in our case, reported no history of any episodes of trauma. The ulcers caused by minor salivary gland tumors such as squamous cell carcinoma, mucoepidermoid carcinoma, and adenoid cystic carcinoma, and Warthin’s tumour usually have an indurated base, tender on palpation and often cause erosive changes on the underlying bone, which did not occur in our case. Cancrum oris (noma) also clinically occurs as an ulcer initially as a gangrenous ulcer on the gingiva, labial mucosa, and hard palate that rapidly spreads with extensive tissue destruction causing extensive facial disfigurement in severely malnourished patients, which was not seen in our case. Immunohistochemical stain helps identify residual myoepithelial cells, as demonstrated by calponin, smooth muscle actin and cytokeratin-7 expression in necrotizing sialometaplasia [9]. Leite M et al. advocated using low-level laser therapy or photobiomodulation to treat necrotizing sialometaplasia [10].

Strength: This was the first case reported in Salem district. Necrotizing sialometaplasia was diagnosed by clinical history and radiological evaluation by the latest maxillofacial cone beam computed tomography imaging (Carestream CS9600 CBCT machine). The cone beam computed tomography has a good submillimeter resolution of about 0.125 mm slice thickness, which helps to provide a clear picture of the anatomic area of interest and a low radiation dose of only (1025 μSv) when compared to computed tomography. The timely and correct diagnosis with the advent of radiological evaluation by the latest advancement in maxillofacial radiology (cone beam computed tomography) by oral medicine specialists prevented unwanted biopsies or surgical procedures in this case. 

The healing time was also rapid, within a short period of about three days, unlike the 4-8 weeks reported in literature also aided in the prevention of performing an unnecessary biopsy or surgical procedures on the patient.

Limitation: Special immunohistochemical staining for demonstrating residual myoepithelial cells was not done in this case.

Patient Perspective: The patient was alleviated from cancerophobia, as he had the habit of chronic smoking in the form of bidi. Also, unwanted biopsy and surgical procedures were prevented in the patient by early prompt diagnosis by oral medicine experts.

## Conclusions

Thorough knowledge and understanding of the controversial etiologies for necrotizing sialometaplasia are essential for oral physicians. The diagnosis of necrotizing sialometaplasia is challenging for oral physicians. Increased awareness is needed for oral physicians to diagnose necrotizing sialometaplasia, which is usually a self-limiting disorder and heals by itself without any treatment. The importance of oral medicine specialists in diagnosing necrotizing sialometaplasia helps to overcome cancerophobia and unnecessary anxiety elicited in the patient. Unwanted biopsy or surgical procedures also can be prevented by the proper diagnosis of necrotizing sialometaplasia. Patients affected by necrotizing sialometaplasia must be educated about the harmful effects of tobacco, and more awareness program on tobacco cessation is essential for creating awareness and prevention of such harmful habits of tobacco among the public. 
